# Foramen magnum meningiomas: a systematic review and meta-analysis

**DOI:** 10.1007/s10143-021-01478-5

**Published:** 2021-01-28

**Authors:** Luca Paun, Renato Gondar, Paola Borrelli, Torstein R. Meling

**Affiliations:** 1 Department of Neurosurgery, Geneva University Hospitals, Rue Gabrielle-Perret-Gentil 4, 1205 Geneva, Switzerland; 2 Department of Medical, Oral and Biotechnological Sciences, Laboratory of Biostatistics, University “G. d’Annunzio” Chieti-Pescara, Chieti, Italy; 3 Faculty of Medicine, University of Geneva, Geneva, Switzerland

**Keywords:** Surgery, Systematic review, Meta-analysis, Meningioma, Foramen magnum, Classification, Outcome

## Abstract

Foramen magnum meningiomas (FMMs) account for 1.8–3.2% of all meningiomas. With this systematic review and meta-analysis, our goal is to detail epidemiology, clinical features, surgical aspects, and outcomes of this rare pathology. Using PRISMA 2015 guidelines, we reviewed case series, mixed series, or retrospective observational cohorts with description of surgical technique, patient and lesion characteristics, and pre- and postoperative clinical status. A meta-analysis was performed to search for correlations between meningioma characteristics and rate of gross total resection (GTR). We considered 33 retrospective studies or case series, including 1053 patients, mostly females (53.8%), with a mean age of 52 years. The mean follow-up was of 51 months (range 0–258 months). 65.6% of meningiomas were anterior, and the mean diameter was of 29 mm, treated with different surgical approaches. Postoperatively, 17.2% suffered complications (both surgery- and non-surgery-related) and 2.5% had a recurrence. The Karnofsky performance score improved in average after surgical treatment (75 vs. 81, *p* < 0.001). Our meta-analysis shows significant rates of GTR in cohorts with a majority of posterior and laterally located FMM (*p* = 0.025) and with a mean tumor less than 25 mm (*p* < 0.05). FMM is a rare and challenging pathology whose treatment should be multidisciplinary, focusing on quality of life. Surgery still remains the gold standard and aim at maximal resection with neurological function preservation. Adjuvant therapies are needed in case of subtotal removal, non-grade I lesions, or recurrence. Specific risk factors for recurrence, other than Simpson grading, need further research.

## Introduction

Intracranial meningiomas account for 25 to 40% of all primary tumors of the central nervous system [30, 38, 43]. About 30% are diagnosed incidentally, while the remaining part is frequently detected when a compression of adjacent neural structures becomes symptomatic [34]. Whereas microsurgical resection is the gold standard for the treatment of meningiomas [32, 50–52], radiotherapy (RT) or stereotactic radiosurgery (SRS) may be considered for patients who are not surgical candidates, for deep tumors, or for atypical meningiomas either after subtotal resection or after recurrence [39, 40].

Foramen magnum meningiomas (FMMs) are skull base meningiomas that account for 1.8 to 3.2% of all meningiomas [1, 5, 15, 47, 49, 51]. They arise from the arachnoid layer at the craniocervical junction, a region defined anteriorly between the lower third of the clivus and the upper margin of C2 body, laterally from the jugular tubercle to the upper margin of C2 lamina and posteriorly from the anterior edge of the squamous occipital bone to the spinous process of C2. The insertion on the dura allowed Bruneau and George [17, 26] to classify FMM as anterior if insertion is on both sides of the anterior midline, lateral if insertion is between the midline and the dentate ligament, or posterior.

FMMs are prone to develop multiple neurological deficits, both pre- and postoperatively [23, 53, 59, 65, 76, 82], due to the neighboring skull base neural and vascular structures, like the V3 and V4 segments of the vertebral artery, the cranial nerves IX–XII, the posterior inferior cerebellar artery (PICA), and the brainstem. These anatomical relationships can be challenging to approach, as the majority (> 80%) of FMMs arise from the anterior or anterolateral aspect of the foramen magnum, i.e., anterior to the dentate ligament [18, 27, 28].

Aside the location and anatomical boundaries, decision making, and management are also influenced by their histological grading, chronologic behavior, and patients’ age, health status, and comorbidities [30]. In some cases, a stabilization may be needed when the lesion or the resection itself causes a mechanical instability. For symptomatic FMMs or tumors with documented growth, the primary treatment is surgical resection [45, 50]. The most feasible approaches remain posterior or postero-lateral to the foramen magnum [8, 17, 25], as anterior approaches have a higher risk of meningitis, neurological morbidity, or mechanical instability [16, 29, 41, 61, 65, 76]. On the other hand, posterior or postero-lateral approaches also carry risks to the brainstem, cranial nerves, and vessels.

With this systematic review and multivariate analysis, our goal is to detail the epidemiology, clinical features, surgical aspects, and clinical outcomes after surgery for FMMs. Once the state of affairs is better described, we will proceed to a description of a multicenter prospective cohort, focusing on potential knowledge gaps identified.

## Methods

### Search strategy, inclusion criteria, and study selection

**Fig. 1:**
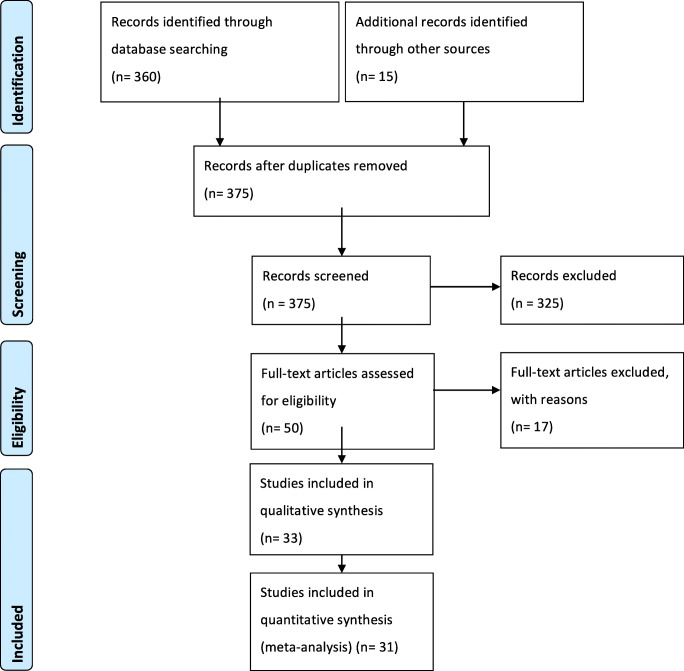
PRISMA-P flow-chart and search strategy

This study protocol followed the Preferred Reporting Items for Systematic reviews and Meta-Analyses (PRISMA-P) 2015 guidelines [70]. No registration was needed. We conducted a restricted search using the keywords (Meningioma AND Foramen Magnum) OR (Meningioma AND Cranio-vertebral Junction) on April 06, 2020 of the following databases: Embase, Cochrane Library, PubMed, and Google Scholar. This resulted in a list of 360 references. In addition, 15 other potentially relevant studies were marked after analysis of the selected references. The first two authors (LP and RG) independently screened all titles and abstracts, and full-text copies of all relevant articles were obtained. In case of a discrepancy, the senior author (TRM) arbitrates until a consensus among the authors was reached (Fig. 1).

The following inclusion criteria were used: (1) Case series, mixed series, or retrospective observational cohorts on FMMs with description of the surgical technique; (2) samples of at least 10 meningioma patients; (3) studies written in the English and French; (4) studies published since 1990, as the standards of micro-neurosurgery has significantly improved since then and results before this era are not comparable [52].

In total, 375 abstracts were screened, and 50 papers were retained for full manuscript screening. Fourteen articles did not present enough data to meet the inclusion criteria (case series or retrospective cohorts with less than 10 meningioma); one article was written in Spanish, another was a review, and lastly, the article from Bertalanffy et al. [10] included cases operated during the 1980s and presented insufficient data on demographics or outcomes.

### Risk of bias and quality of studies

The accepted articles were independently graded by one author (LP) according to the Newcastle–Ottawa Quality Assessment Scale for quality assessment of non-randomized studies [80]. The level of evidence for each study was evaluated using the Oxford Centre for Evidence Based Medicine guidelines [58].

### Data collection

The two first authors (LP and RG) extracted the data independently. Data extracted included the following items: (1) study ID; (2) study characteristics (author, year, country, type of study); (3) patient demographics; (4) sample size; (5) mean maximal lesion dimension; (6) preoperative and postoperative Karnofsky Performance Status (KPS); (7) lesion location (anterior, lateral, or posterior); (8) preoperative surgery and/or RT; (9) World Health Organization (WHO) meningioma grade; (10) recurrence; (11) surgical technique (including surgical approach, vertebral artery (VA) transposition, jugular tuberculum resection, mastoidectomy, number and extent of occipital condyle (OC) resection, cervical instability, and eventual need for fixation [48]); (12) neurological outcome (improved, unchanged or worsened); (13) Simpson [74] resection grade (gross total removal (GTR) if Simpson grades I and II, and subtotal resection (STR) if Simpson grades III and IV); (14) postoperative complications; (15) postoperative morbidity (transient or permanent); (16) postoperative mortality; (17) postoperative follow-up (FU) time; (18) postoperative RT or stereotactic radiosurgery (SRS); and (19) overall survival (OS).

### Statistical analysis

Results for continuous variables are reported as mean ± standard deviation (SD) or range. For articles that did not report mean and SD, we estimated the mean and SD according to the methodology described by Hozo et al. [33]. Categorical variables are presented as median and quartiles or by absolute and relative frequencies.

A meta-analysis was performed, firstly by excluding selection bias through an Egger’s test for small-study effects. Subsequently, a random effects-model was used to search for a correlation between meningioma characteristics (surgeon/center, location, and size) and GTR rate.

## Results

### Patient demographic results

From 1996 to 2020, we considered 33 retrospective or case series studies (Table 1). No prospective cohorts were found. One thousand fifty-three patients were included, with a mean study sample size of 33 patients (Table 1). As expected from meningioma epidemiology, females were found to be more affected (*N* = 567; 53.8%) than males (*N* = 272; 25.8%). For 214 patients, the gender was not stated. The mean age was of 52.4 years with a range of 10 to 81 years (Table 1). Forty-four patients already benefited from a precedent FM surgery and 31 had RT before surgical resection (Table 1).

**Table 1:** Summary of all included studies on FMM with follow-up data

Study design	Retrospective Case series
Number of patients	Total: 1053 Mean: 33
Age (years)	Mean: 52.4 years Range: 10–81
Gender	Not stated: 214 Female: 567 Male: 272
Localization (%)	Anterior: 65.6 Lateral: 21.6 Posterior: 12.8
Maximal diameter (mm)	Mean: 29 Range: 3–89.5
KPS preoperative	Mean: 75
WHO grade (*N*)	Grade I 318, grade II 10, grade III 1, NA 724
Preoperative surgery (*N*)	44
Preoperative radiotherapy (*N*)	31
Recurrence (%)	2.5
Mean resection (%)	GTR (Simpson I and II): 80 STR (Simpson III and IV): 20
Postoperative complication (%)	17.2
Follow-up (months)	Mean 51 Range 0–258
KPS postoperative	Mean: 81 Range: 0–100
Postoperative radiotherapy (*N*)	43

### Meningioma characteristics

Most of the 1053 surgically treated meningiomas were located in the anterior part of the foramen magnum (65.6%). In two studies, the exact location was not detailed. The mean maximal diameter was 29.1 mm (range 3–89.5) with most of the lesions benign, i.e., WHO grade I. Information regarding WHO grade was lacking for 724 (68.8%) meningiomas (Tables 2 and 3).

**Table 2:** Overall patient demographics, meningioma characteristics, and preoperative status

Year, author	Sample size (*n*)	Gender (*n*, M/F)	Mean age (years ± SD, range)	Location (%)			KPS (mean ± SD)		Mean maximal diameter (mm ± SD, range)	VA encasement (%)	Instability
				Ant	Lat	Post	Preop	Postop			
1996, Samii [67]	38	25/13	49	95		5	66	64	NA	40	0
1997, George [26]	40	11/29	51.6 (14–76)	45	52.5	2.5	NA	NA	13 (3–25)	38	NA
1999, Sharma [71]	10	NA	41 (14–75)	50		50	NA	NA	NA	NA	Yes
1999, Salas [66]	24	NA	NA	100			74.7 ± 4.69	76.4 ± 4.33	35 (10–56)	NA	0
2000, Arnautovic [5]	18	5/13	58 (36–77)	100			70	85.5	NA	NA	0
2001, Roberti [65]	21	14/28	47 (10–81)	100			80.2 (40–100)	65 (40–80)	31 (5.3–89.5)	NA	NA
2001, Goel [29]	17	6/11	39.2 (17–72)	100			NA	NA	31.4 (21–38)	59	0
2002, Bertalanffy [9]	25	NA	NA	64	28	8	NA	NA	NA	NA	NA
2003, Boulton [15]	10	2/8	55 (34–72)	60	10	30	NA	NA	NA	NA	NA
2004, Wang [79]	11	4/7	49 (16–69)	100			NA	NA	(21–40)	NA	NA
2004, Pamir [59]	22	4/18	47 (18–74)	91		9	73	94	NA	40	0
2005, Margalit [46]	18 (42)	14/28	47 (14–80)	100			NA	NA	34 (21–59)	NA	NA
2006, Bassiouni [8]	25	6/19	59.2 (33–78)	32	57	11	79 (50–90)	89 (30–100)	29 (18–43)	43	NA
2006, Shin[72]	16	NA (16/30)	41.1 (8–76)	NA	NA	NA	NA	NA	NA	NA	0
2009, Wu [82]	114	46/68	52.3 (28–76)	70.2	21.1	8.8	72.5 ± 8.3	83.5 ± 8.6	33.5 (15–47)	40.4	NA
2009, Kandenwein [35]	16	4/12	61 (40–85)	81.3	12.5	6.3	NA	NA	(20–60)	NA	0
2009, Borba [14]	15	1/14	55.9 (42–74)	53.3	46.7		NA	NA	27 (20–50)	NA	NA
2010, Kano [36]	23	8/15	56 (26–70)	39.1	60.9		NA	NA	25.9 (12–50)	NA	0
2010, Cusimano [21]	20	NA	NA	55	25	20	NA	NA	NA	50	0
2010, Bruneau [18]	107	NA	NA	39.4	54.8	5.8	NA	NA	NA	NA	NA
2012, Talacchi [76]	64	16/48	59 (27–82)	37.5	62.5		> 70 (34), 60–70 (11) e < 60 (19)	NA	35	48	NA
2013, Lynch [44]	12	3/9	48.3 (33–61)	91.6		8.4	NA	NA	35.1 (21–48)	Y	NA
2014, Colli [19]	13	2/11	54.15 ± 15.4 (28–77)	38.5	53.8	7.7	> 80 (9)	> 80 (5)	25.6	Y	NA
2015, Moscovici [56]	33	NA (12/32)	NA 52 (14–77)	100			NA	NA	NA	NA	NA
2016, Tao [77]	26	NA (19/30)	48.6 ± 13.3	38.8	61.2		NA	< 80 in 5 (10.2) e > 80 in 44 (89.8)	30 (10–64)	NA	NA
2016, Park [60]	16	NA (6/22)	NA (48.9) (22–69)	100			NA (77 (60–100))	NA (78 (0–100))	30 (17–43)	Y	NA
2016, Yamahata [83]	16	3/13	58.4 ± 11 (38–77)	NA	NA	NA	76.25	93.75	28 ± 7.5 (17–48)	Y	0
2016, Dobrowolski [23]	24	6/18	52 (10–82)	12.5	16.7	70.8	85 (70–100)	NA	25.08	Y	NA
2017, Li et al. [41]	185	61/124	49.4 ± 11.5	65.9	26.5	7.6	80	80	33 ± 7	Y	NA
2017, Bocchetti [13]	14	4/10	64.5 (55–77)	42.9		57.2	NA	NA	16.07	No	NA
2019, Bilgin [12]	11	3/8	60.8 (32–75)	36.3	18.2	45.5	72.72	84.54	NA	NA	NA
2019, Giordano [28]	39	16/23	53 ± 14 (15–78)	84.6		15.4	NA	NA	31.1 ± 10.7	Y	NA
2019, Magill [45]	28	8/20	57.2 (30.6–74.4)	54	28	18	NA	NA	30 (12–47)	Y	NA

*M*, male; *F*, female; *SD*, standard deviation; *KPS*, Karnofsky performance status; *VA*, vertebral artery

**Table 3:** Surgical approaches and strategies, meningioma histology, and outcomes

Year, Author	WHO grade			Surgical approach	CR (%)	Extent CR	Outcome* (%)			Resection (%)		Morbidity rate (%)		Mortality rate (%)	Recurrence (% during FU)	Postop RT (*N*)	Follow-up (months)
	I	II	III				↑	=	↓	GTR	STR	Transient	Permanent				
1996, Samii [67]				PM, LSO	17.5	1/3				63	30	37	5	6	5	No	21
1997, George [26]				PLA, ALA, MPA	100	Partial	90	2.5	7.5	87.5	10	NA	0	7.5	0	No	57.6
1999, Sharma [71]				PM, FL	0.0	0	70	0	30	100		NA	NA	15	NA	NA	NA
1999, Salas [66]				TC/ELTJ	100	1/3	NA	NA	NA	66	33	NA	NA	0	0	NA	14.8
2000, Arnautovic [5]	18	0	0	TC/ELTJ	100	½–1/3	89	11	0	75	12.5	55	11.1	16.6	5.5	4	40
2001, Roberti [65]				EL TC	NA	2/3–2/4	NA	NA	NA	76	24	NA	21.5	9.5	NA	No	19.3
2001,Goel [29]				SO	11.8	1/3–1/4	100			82	18	60	6	NA	0	NA	43
2002, Bertalanffy [9]				SO	NA	NA	NA	NA	NA	NA	NA	NA	NA	NA	NA	1	NA
2003, Boulton [15]				SO	0	0	70	20	10	90	10	40	10	0	10	NA	33
2004, Wang [79]				NA	NA	NA	6	NA	2	64	36	NA	NA	0	NA	1	NA
2004, Pamir [59]	21	1	0	SO, FL	95	1/3	NA	NA	NA	95.5	4.5	27	4.5	0	0	NA	40
2005, Margalit [46]				Lat	50.0	Partial	NA	NA	NA	61.1	38.8	NA	NA	5.5	0	NA	NA
2006, Bassiouni [8]	23	2	0	FL	0.0	0	88	NA	NA	96	4	40	8	4	0	NA	73.2
2006, Shin [72]				EL	43.7	1/3	NA	NA	NA	NA	NA	NA	NA	NA	NA	NA	66.1
2009, Wu [82]				PM, FL, EFL	0.9	1/3–1/2	NA	NA	NA	86	14	NA	NA	1.8	0.9	NA	90.3
2009, Kandenwein [35]				SO	18.7	1/3	50	31.2	12.5	87.5	12.5	18.75	43.75	6.3	NA	NA	43.5
2009, Borba [14]				Lat	53.3	1/32/3	NA	NA	NA	80	13.3	6.7	6.7	0	0	NA	23.6
2010, Kano [36]				SO, TC, Transpetr	NA	NA	NA	NA	NA	65.2	34.7	30.4	17.4	0	4.3	1	42.8
2010, Cusimano [21]				EL, PM, SO	0	0	75	10	15	75	25	40	10	0	0	NA	33.1
2010, Bruneau [18]				FL, AL, SO	NA	NA	NA	NA	2.8	86	11	NA	NA	1.8	8.4	NA	NA
2012, Talacchi [76]				SO	NA	NA	27	16	54	81	19	91	9	0	1.5	3	138
2013, Lynch [44]	12	0	0	SO	0	0			41.6	83.3	16.6	NA	NA	16.6	8.3	1	98.4
2014, Colli [19]	13	0	0	FL	30.76	1/3	7.6	53.84	30.76	69.2	30.8	38.5	7.7	7.7	7.8	NA	47.31
2015, Moscovici [56]				Modified FL	NA	1/3	NA	NA	NA	48	22	11.4	6.8	0	NA	NA	NA
2016, Tao [77]				SO, FL	NA	NA			22.4	85.7	14.3	NA	NA	4.1	4.1	NA	40.18
2016, Park [60]				SO	100	< 1/3			28.6	93.8	6.3	NA	14.2	0	0	NA	50.4
2016, Yamahata [83]				FL, TC	25	NA			25	93.8	6.3	NA	25	NA	NA	NA	NA
2016, Dobrowolski [23]	NA	NA	1	MSO	8.3	1/3	NA	NA	NA	80	20	NA	8	NA	4.17	NA	45.6
2017, Li et al. [41]	180	5	0	SO, EFL, FL RC, FLTC	Y	NA		62.7	37.3	83.2	16.8	28.6	14.1	NA	7.2	24	110.3
2017, Bocchetti [13]	14	0	0	SO	NA	NA	NA	NA	NA	100		14.28	NA	0	0	NA	24
2019, Bilgin [12]	11	0	0	SO, FL	36	1/3	NA	NA	NA	82	18	18.8	NA	0	0	NA	18
2019, Giordano [28]				SO, FL	NA	NA	NA	NA	NA	74.4	25.6	NA	NA	0	0	NA	NA
2019, Magill [45]	26	2	0	SO, FL	1/3	NA	63	NA	NA	39	61	NA	NA	0	3.6	NA	70.8

*(each study used different scales to measure outcome (GOS, modified Rankin Score, among others)

*WHO*, World Health Organization; *CR*, condyle resection; *GTR*, gross total resection; *STR*, subtotal resection; *FU*, follow-up; *RT*, radiotherapy; *PM*, paramedian; *LSO*, lateral suboccipital; *PLA*, posterolateral approach; *ALA*, anterolateral approach; *MPA*, midline posterior approach; *FL*, far-lateral; *TC*, transcondylar; *ELTJ*, extreme-lateral transjugular; *TC*, transcondylar; SO, suboccipital; *Lat*, lateral; *EFL*, extended far-lateral; *Transpetr*, transpetrosal; *MSO*, midline suboccipital; *RC*, retrocondylar

Aside from meningiomas themselves, other anatomical relationships and mechanical consequences were in some cases meticulously described. Vertebral artery encasement was mentioned in 8 of 33 studies and was found in at least 178 patients (40.0%) (Table 2). A mechanical instability is a possible complication from tumoral bone invasion or from partial or complete occipital condyle resection [44], but it was not possible to quantify the rate due to imprecise data reporting (Table 2).

### Surgical approaches and anatomical challenges

A large variety of surgical approaches were used both inter- and intra-institutionally (Table 3). The approaches were cited, but no quantitative data was given in most of the studies. Among the preferred ones were the far lateral (FL) [24, 37, 68, 71, 78], modified far lateral (modified FL) [56, 75], Eextended far lateral (EFL) [37], extreme lateral (EL) [3, 6, 37, 42, 60, 66, 73, 78], lateral (Lat) [14, 46], suboccipital (SO) [9, 11, 13, 19, 21–23, 29, 35, 78, 79], transcondylar (TC) [2, 7, 36, 62, 64], transoral (TO) [4, 20, 63], or transpetrosal [54] with small technical variations also described (Table 3).

The occurrence or extent of condyle resection was frequently not stated, but in four cohorts (12.1%), some degree of condyle resection was performed in all patients; in seven cohorts (21.2%), condyle resection was performed in at least half of patients, and in 11 cohorts (33.3%), less than one-third of the condyle was resected, whereas in 4 cohorts (12.1%), a maximum of two-thirds of condyle mass was resected unilaterally (Table 3). To our knowledge, no cranio-cervical fixation was performed.

### Outcomes and recurrence

The mean follow-up was of 51 months, with a range of 0–258 months (Tables 1 and 2). 2.5% had a recurrence. Forty-three (4.1%) patients had postoperative RT. The mean preoperative KPS was of 75, with a slight improvement into a mean of 81 (*p* < 0.001) after surgical treatment. Mortality rates ranged from 0 to 16.6% (Table 3). Morbidity was classified as transient or permanent depending on its presence at the end of clinical follow-up (Table 3).

Postoperatively, 17.2% (range 0–91) of the patients suffered complications (both surgery-related and non-surgery-related). Surgical outcomes were trichotomized into clinical improvement, stability, or deterioration. This compromise was made because of the vast heterogeneity of different outcome scales used in the considered studies. Among the most commonly used scales, we find Glasgow Outcome Scale (GOS) and modified Rankin Score (mRS) (Table 3). Only 16 studies reported outcomes, and most of these (*N* = 11) had more cases with postoperative clinical improvement than worsening (Table 3).

**Fig. 2:**
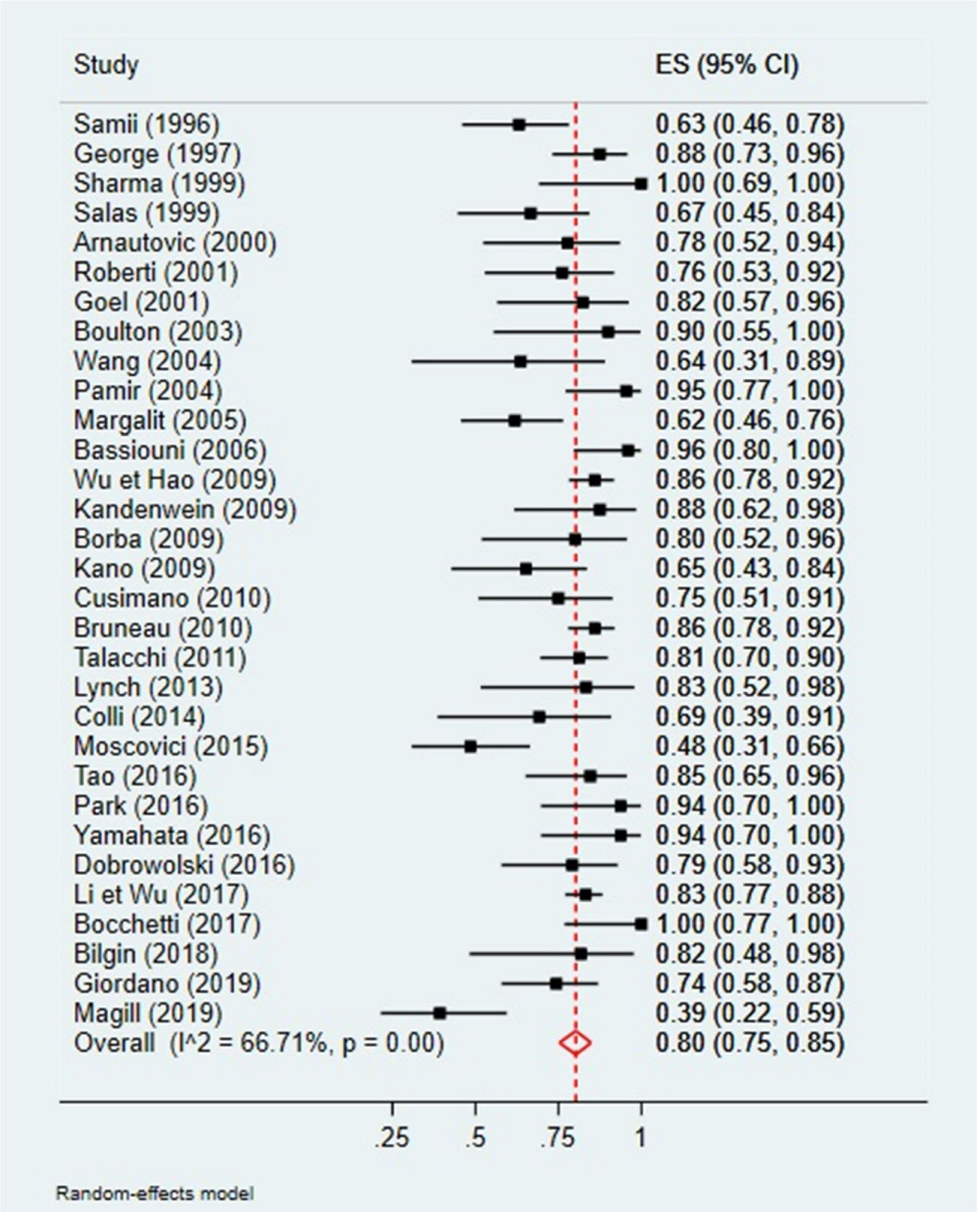
Quantitative analysis with a forest plot representation of GTR according to FMM location (≤ 50% or > 50% of meningiomas located anteriorly). GTR, gross total resection; FMM, foramen magnum meningioma

**Fig. 3:**
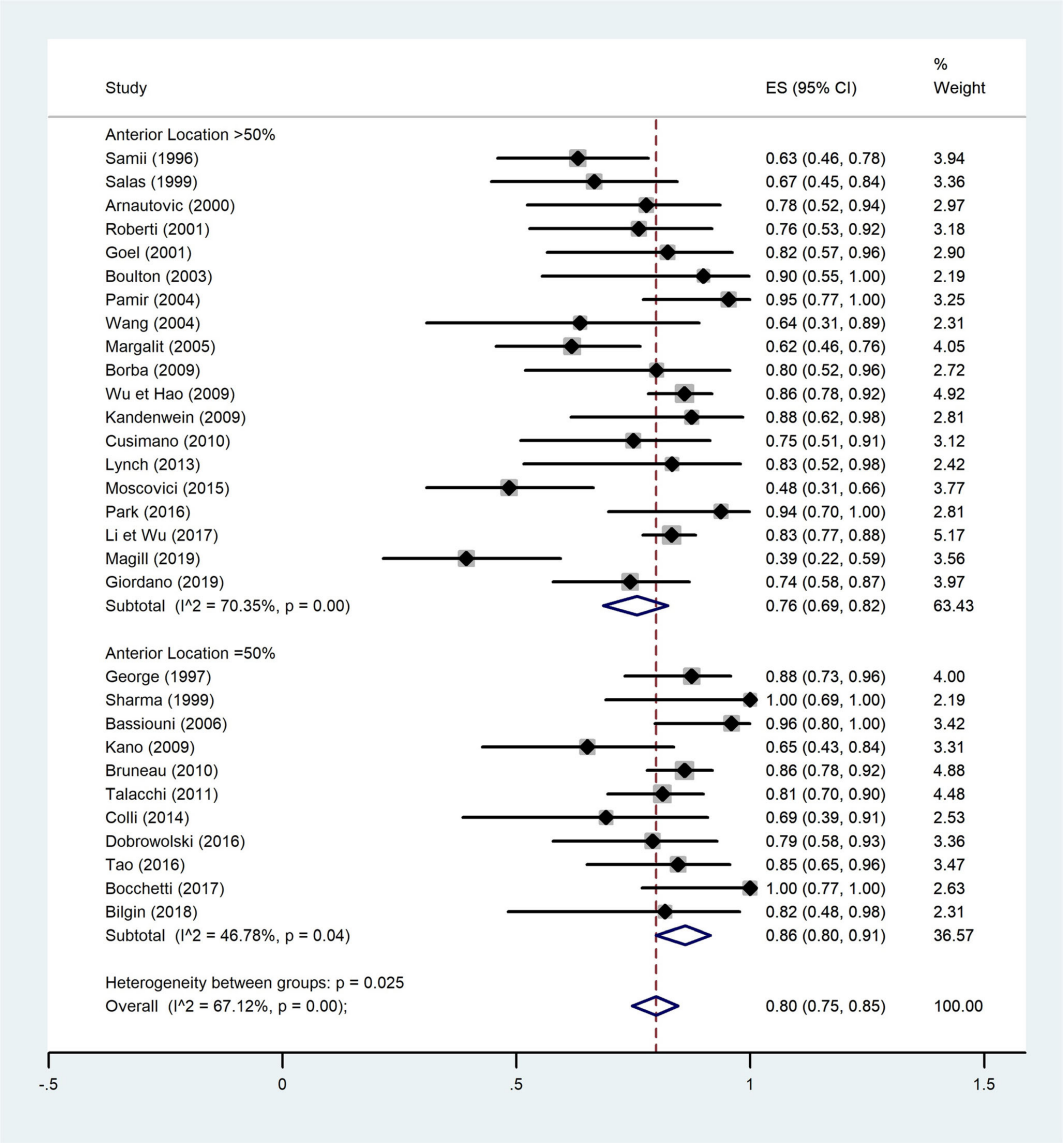
Quantitative analysis with a forest plot representation demonstrating a significant GTR rate for studies with predominantly non-anterior FMM. GTR, gross total resection; FMM, foramen magnum meningioma

**Fig. 4:**
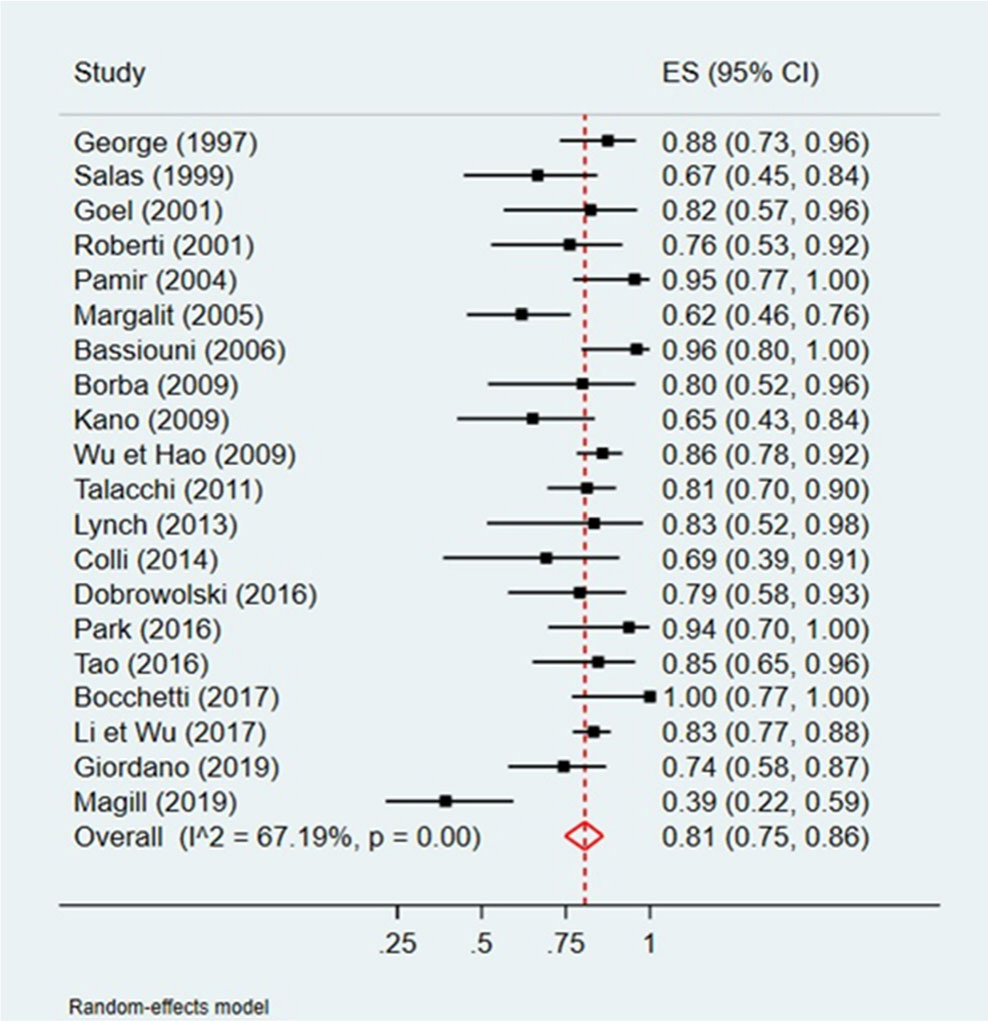
Meta-analysis of GTR by tumor-to-foramen magnum ratio with a forest plot representation. The analysis used FMM mean maximal diameter. We divided the studies in group 1 (ratio between 0 and 25% included), group 2 (ratio 25–30% included), and group 3 (ratio superior to 30%). GTR, gross total resection; FMM, foramen magnum meningioma

**Fig. 5:**
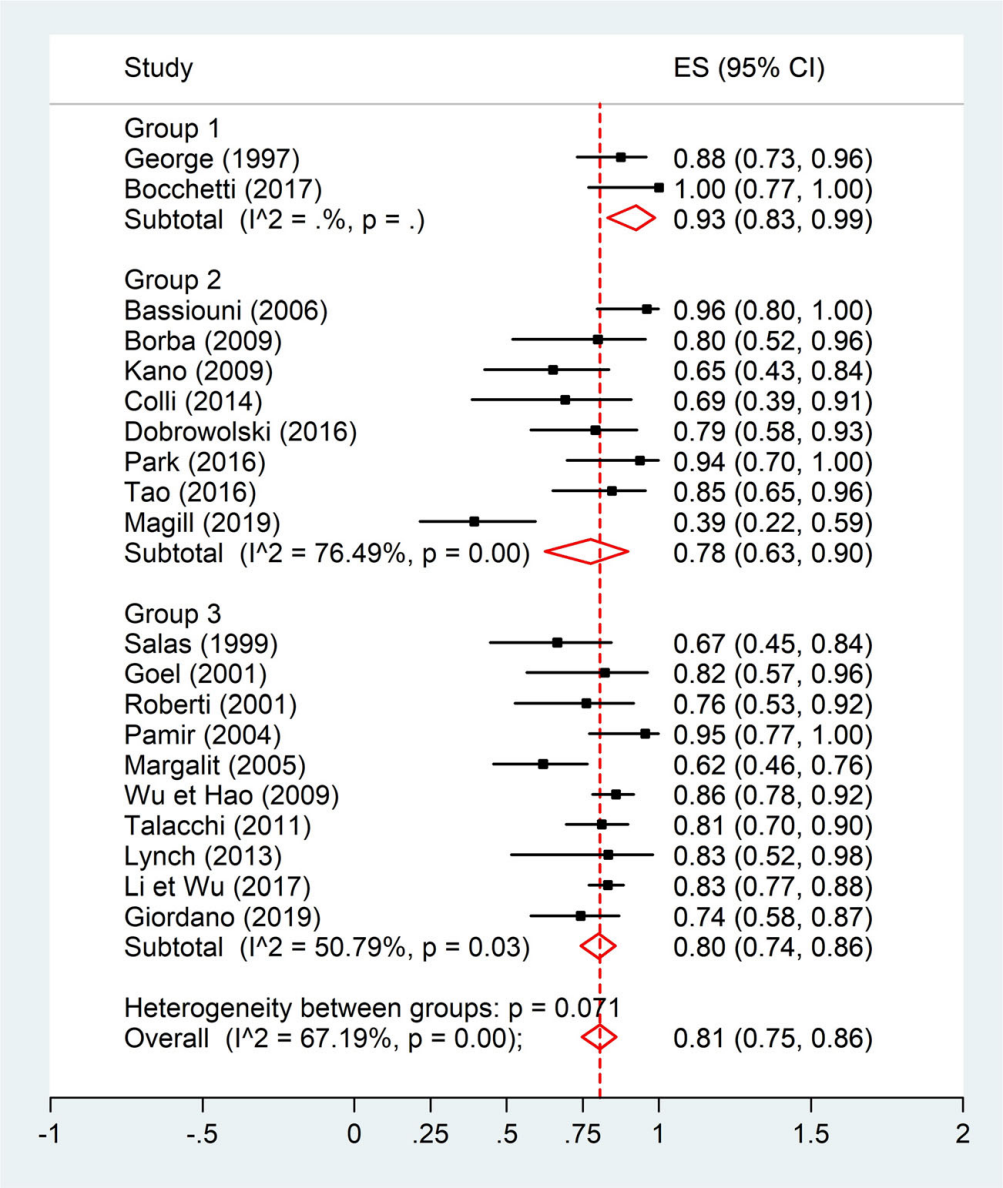
Meta-analysis of GTR by mean maximal meningioma diameter with a forest plot representation. FMM mean diameter was divided into 3 subgroups (group 1 (0–25 mm), group 2 (> 25 and ≤ 30 mm) and group 3 (> 30 mm). GTR, gross total resection; FMM, foramen magnum meningioma

A quantitative analysis was conducted to assess a potential correlation between GTR (Simpson I and II) and tumor location (anterior or non-anterior) or GTR and tumor maximal diameter (Figs. 2, 3, 4, and 5). The first meta-analysis was conducted on 31 studies and showed an important heterogeneity (Fig. 2). We used a random-effects model and divided the study population in two subgroups: ≤ 50% or > 50% meningiomas located anteriorly (Fig. 3). Egger’s test for small-study effects ensured no publication bias *(p* = 0.566). Heterogeneity was higher in studies presenting > 50% anterior FMM (*p* = 0.025). The respective forest plot showed a significant higher rate of GTR (*p* = 0.025) for those cohorts with predominant lateral or posteriorly located FMM (≤ 50% in anterior location), if analyzed separately (Fig. 4). This observation can be explained by an easier access to the tumor when located lateral or posterior to neurovascular components of the FM. The second quantitative analysis included 20 studies and was also limited by a high heterogeneity. Here, the focus was to correlate GTR and the ratio tumor diameter:FM dimension (Figs. 4 and 5). We used a random-effects model and divided the considered population in three subgroups, according to mean meningioma maximal diameter. We divided the studies in group 1 (FMM size between 0 and 25 mm included), group 2 (> 25 and ≤ 30 mm) and group 3 (> 30 mm). Egger’s test confirmed no publication bias (*p* = 0.537). The forest plot showed a significant higher rate of GTR in group 1, i.e. for FMMs smaller than 25 mm (*p* < 0.05).

## Discussion

Our systematic review confirms that foramen magnum meningioma (FMM) is a rare pathology that requires high microsurgical expertise. Clinical series from major centers range from a few cases (5–6) to a maximum of 185, with only three centers having more than 100 cases reported (Table 1). One effect of this limited FMM case load is the difficulty to systematize approaches, strategies, and outcome measurements. This limitation ultimately prevents proper comparison between cohorts and centers and their surgical results in a long-term fashion. Lack of WHO grading, not mentioned in more than half of cases, did not permit a histological analysis nor correlation.

Arising from an intricate anatomical area close to vital functions, FMMs are undoubtfully complex to treat and even the most experienced skull base teams report a relatively high overall mean complication rate of 17.2% (Table 1). However, despite the morbidity inherent to FMM surgery, our analysis shows a significant KPS improvement after surgery of 7 points (*p* < 0.001), which is likely to be an underestimation as the mean follow-up time was short in most of the clinical series. Further observations from this qualitative and quantitative review include (1) - most meningiomas arise from the anterior or lateral wall of the FM; (2) - the available data regarding bony meningioma invasion or condyle resection and long-term clinical (pain) or radiological (C0-C2 translation or dislocation) craniocervical junction instability is scarce [12, 72]; (3) - the follow-up for FMM is too short to allow conclusions about long-term progression-free survival or recurrence (less than 5 years); (4) - on average, patients improve KPS after surgery (*p* < 0.001); (5) - FMM size < 25 mm and non-anteriorly tumor location significantly increases the rate of GTR.

By excluding series prior to 1990, we ensured that only the microneurosurgical era was considered, but there has also been an important trend of lower mortality and morbidity rates of meningioma surgery over the past three decades [52]. Important technological advancements include the optical performance of microscopes, advanced real-time angiography, tumor imaging and augmented reality, as well as angled endoscopes that can help to visualize hidden angles. Also, neuronavigation probably flattened the learning curve with respect to anatomical recognition during surgery. Lastly, neuromonitoring with evoked potentials and cranial nerves mono- or bipolar stimulation and intraoperative function assessment have allowed for safer resections. Mortality rates higher than 10% were mainly observed in the smaller series (Tables 2 and 3), but the mortality rates are still higher than those for meningiomas in other locations [50].

Morbidity remains difficult to separate from complication rate and furthermore lacks distinction between transient and permanent in most series. Tumor-dependent risk factors of increased morbidity include anterior tumor location [27, 67], tumor invasiveness and extradural extension [18], recurrent lesions with adhesions [67], VA encasement [31], absence of arachnoidal sheath [8], and tumor size. The most common preoperative deficits are lower cranial nerves palsies, which tend to recover almost completely after surgery [67], but Samii et al. [67] found lower recovery potentials after *en plaque* meningiomas or recurrent tumors.

With regard to extent of resection, our meta-analysis correlates GTR with FMM size (< 25 mm) and non-anterior location when subdividing and selecting cohorts according to these parameters (Figs. 3 and 5). The question remains whether the average 80.9% of GTR (Simpson I and II) is as reliable in such anatomically rich region as it is in less eloquent and free areas, for instance convexity location [32]. Among the factors preventing GTR, the literature identifies vertebral artery (VA) encasement [67] and extradural extension [27] as independent vectors. For now, no further independent risk factors for subtotal resection of FMM were identified. However, it would be easy to imagine that the preferred surgical approach could be one of these limiting factors if randomization was allowed for such variable. Instead, surgeons’ experience and trust guide this choice.

The most commonly used approaches in the existing literature comprise the far-lateral approach [57, 64, 81], and the extreme-lateral approach [37, 69], also named antero-lateral approach. The former is a lateral suboccipital approach just medial to the occipital condyle and C1 upper facet joint, while the latter is a direct lateral way, anterior to the sternocleidomastoid muscle and between the internal jugular vein and the VA. Both approaches permit drilling of the occipital condyle but result in different angles of approach. The far-lateral approach was the preferred choice of most groups, even for anterior FMMs (Table 3). During this approach, the VA is controlled in the horizontal portion of the V3 segment, above the C1 posterior arch. It can be further divided in retro- or transcondylar, but usually needs less condyle destruction to provide a good exposure. The extreme-lateral approach usually goes partially transcondylar and implies VA transposition and one-third to half occipital condyle and upper C1 facet-joint drilling without any secondary instability described [5]. All in all, the increased surgical corridor and exposure do not seem to be enough to compensate for the risks of accessory nerve dissection, VA dissection or rupture, and instability related with more condyle drilling [17, 77, 83]. Other approaches include the transoral path which is linked with increased risk of CSF fistula and meningitis after crossing of the contaminated oral cavity, poor access to laterally extending tumors resulting in a low rate of complete resection, and increased risk of postoperative instability and velopalatine insufficiency [20, 55]. It is of notice that posterior midline approaches, even if they do not allow a full vascular and neurological tissue control in some specific meningiomas, are still preferred by some authors. This can be explained by their feasibility with less potential approach-dependent complications and with shorter operative time.

Vertebral artery (VA) encasement and its management during surgery remains an anatomically and technically interesting aspect. In general, the reported series failed to present details on the topic. One can probably deduct that VA was often spared and left with some residual tumor, but still little is written on recurrence or need for irradiation in such cases. Similarly, there is a lack of information with respect to mechanical instability in FMM, both from bony invasion and iatrogenic condyle resection. This is an increasingly important subject as it can cause secondary compression through luxation of the cervical spine, neural compression, or chronic headache and neck pain with a major impact in patients’ quality of life and outcome. Authors tend to agree that condyle resection should be, if possible, limited to the destroyed or invaded bone, and stays overall safe if less than half of the C0-C2 joints are resected [25].

Over the last 30 years, the treatment paradigm for meningiomas has changed. Instead of aiming for complete tumor resection at all cost, tumor reduction surgery within the best secure margins is often preferred nowadays [30, 39]. This paradigm shift follows a tendency also seen for other central nervous system tumors with no harm for progression-free survival. Also, the targeting of stereotactic radiosurgery has become more accurate when compared with old external beam radiation devices, probably opening a window for safer irradiation while protecting the neighboring structures. Lastly, proton-beam therapy is also a potentially interesting technique that remains underreported for FMM.

It is important to centralize FMM treatment in referral centers, permitting neurosurgeons to be exposed to an adequate specific surgical volume. This facilitates an appropriate training, independently from the surgical approach, resulting in a lower rate complication and morbidity and increased extent of safe tumor resection.

## Conclusion

FMM is a challenging and rare pathology that has to be considered from a multidisciplinary point of view. At the moment, surgery remains an essential procedure to obtain tissue and to reduce brainstem compression and edema. If in the past surgery was considered the panacea of this disease, nowadays, surgery should be considered a “primum inter pares” tile in the treatment process, concentrated in highly specialized referral centers, where radiotherapists, geneticists, and oncologists should help to give to the patient the best possible quality of life with the maximal resection and without compromising neurological and vascular function.

## Data Availability

Not applicable—only review of literature
